# Metabolic Insights Into Infochemicals Induced Colony Formation and Flocculation in *Scenedesmus subspicatus* Unraveled by Quantitative Proteomics

**DOI:** 10.3389/fmicb.2020.00792

**Published:** 2020-05-07

**Authors:** Sebastiana Roccuzzo, Narciso Couto, Esther Karunakaran, Rahul Vijay Kapoore, Thomas O. Butler, Joy Mukherjee, Erika M. Hansson, Andrew P. Beckerman, Jagroop Pandhal

**Affiliations:** ^1^Department of Chemical and Biological Engineering, University of Sheffield, Sheffield, United Kingdom; ^2^Centre for Applied Pharmacokinetic Research, The University of Manchester, Manchester, United Kingdom; ^3^Department of Biosciences, College of Science, Swansea University, Swansea, United Kingdom; ^4^Department of Animal and Plant Sciences, University of Sheffield, Sheffield, United Kingdom

**Keywords:** *Daphnia* infochemicals, *Scenedesmus*, induced defenses, flocculation, colony formation, physiological ecology, iTRAQ proteomics

## Abstract

Microalgae can respond to natural cues from crustacean grazers, such as *Daphnia*, by forming colonies and aggregations called flocs. Combining microalgal biology, physiological ecology, and quantitative proteomics, we identified how infochemicals from *Daphnia* trigger physiological and cellular level changes in the microalga *Scenedesmus subspicatus*, underpinning colony formation and flocculation. We discovered that flocculation occurs at an energy-demanding ‘alarm’ phase, with an important role proposed in cysteine synthesis. Flocculation appeared to be initially stimulated by the production of an extracellular matrix where polysaccharides and fatty acids were present, and later sustained at an ‘acclimation’ stage through mitogen-activated protein kinase (MAPK) signaling cascades. Colony formation required investment into fatty acid metabolism, likely linked to separation of membranes during cell division. Higher energy demands were required at the alarm phase, which subsequently decreased at the acclimation stage, thus suggesting a trade-off between colony formation and flocculation. From an ecological and evolutionary perspective, our findings represent an improved understanding of the effect of infochemicals on microalgae-grazers interactions, and how they can therefore potentially impact on the structure of aquatic communities. Moreover, the mechanisms revealed are of interest in algal biotechnology, for exploitation in low-cost, sustainable microalgal biomass harvesting.

## Introduction

*Scenedesmus* spp. have predominantly been isolated in freshwater bodies and have also been found in soils all over the world (Trainor, 1998). They are easily cultivated in the laboratory and can tolerate a wide range of environmental conditions, making them ideal candidates for ecological, evolutionary and biotechnology research (Lürling, 2003). In response to several forms of environmental stresses, numerous microalgal species can produce colonies, called *coenobia* and/or produce aggregates, also referred to as flocs. Colony formation and aggregation/flocculation are interesting cellular processes for several reasons. First, colony formation is typically interpreted as an altered cell division process leading to multicellular entities with a common mother cell wall (Bisova and Zachleder, 2014). In contrast, aggregation defines a process of adhesion among existing dispersed cells (Li et al., 2015) and likely involves the production of extracellular polymeric substances (EPS) that ‘glue’ single cells together. Second, as defense responses, their perceived role is to increase survival, and this has implications for the structure of ecological communities in freshwater (and soil) habitats. Finally, aggregation/flocculation is of great interest in microalgal biotechnology, where it is a vital aspect of the harvesting process for generating value-added products (Vandamme et al., 2013; Wan et al., 2015; Ummalyma et al., 2017). Methods that avoid addition of chemicals or expensive polymeric flocculants are particularly attractive (Zhu et al., 2017; Malik et al., 2019; Okoro et al., 2019). Thus, elucidating and understanding the mechanisms inducing colony formation and aggregation/flocculation is critical in fundamental and applied fields (Ghosh and Das, 2015; Alam et al., 2016, 2017; Zhu et al., 2016; Rashid et al., 2018). There are many known triggers to colony formation and floc formation. Metal salts and biopolymers are known to aggregate/flocculate microalgae, with mechanisms ranging from ionic suppression to bridging (Uduman et al., 2010). Zooplankton grazers of microalgae are not only known to induce aggregation/flocculation, but also colony formation. This is often termed infochemical flocculation. This response of the algae is understood to be triggered by products excreted by the zooplankton grazers (Hessen and Vandonk, 1993; Lampert et al., 1994; Lürling and vanDonk, 1996) and has been reported to be induced only by herbivorous zooplankton “chemical cues” (Lürling, 2003).

A summary of the current literature reports on characterization of infochemicals is provided in Table 1. A meta-analysis of existing studies highlighted grazer-specific effects that can reach similar magnitudes to metal salts and suggested that a clear distinction between *coenobia* formation and aggregation-based mechanisms is necessary (Roccuzzo et al., 2016). This knowledge has largely arisen from experiments measuring dose-dependent production of colonies and aggregates, but we still know too little about how infochemicals trigger the physiological and cellular level changes that underpin colony formation and flocculation. This is further complicated by very few studies on the actual identity of infochemicals. There are several reasons for this. First, infochemicals activity might be the result of several compounds that act synergistically, in which case, bioassays are unsuitable to detect a response after the purification of individual components. Second, the lack of bioassays that are robust and resistant to confounding effects makes the characterization of infochemicals tedious and can result in false positive identifications. Finally, bioassay-guided identification is highly time consuming (Pohnert et al., 2007; Saha et al., 2019). In addition to experiments measuring dose-dependent production of colonies and aggregates, ‘omics-based approaches’ (i.e., genomics, transcriptomics, and proteomics) have the potential to reveal how these processes are triggered and regulated intracellularly, providing insight from an ecological, evolutionary and biotechnology perspective. To date, few omics-based approaches have been applied to uncover metabolic mechanisms linked to infochemicals induced flocculation and colony formation (Gulez et al., 2014; Poulson-Ellestad et al., 2014; Schmid et al., 2015; Yu et al., 2016; Harke et al., 2017). Harke et al. (2017) performed a transcriptomic study to elucidate the response of the cyanobacterium *Microcystis* to direct and indirect exposure to *Daphnia.* Bloom forming and toxic *Microcystis* cells are known to harbor the metabolic capability to hinder rates of zooplankton grazing. They reported higher transcription of genes related to secondary metabolites with putative roles in defense against grazing (e.g., microcystin peptide synthesis genes), heat shock proteins and photosynthetic processes, the latter indicating grazer-induced stimulation of energy acquisition pathways. In addition, gene transcripts associated with production and secretion of polysaccharides (i.e., *tag*H, *rfb*B, *rfb*C, and *rfb*D) significantly increased in abundance upon exposure to infochemicals and were linked to colony formation of *Microcystis* as a defense against grazing (Harke et al., 2017). Similarly, Amato et al. (2018) reported activation of stress-related genes in the chain-forming diatom *Skeletonema marinoi* when exposed to grazer cues. They also observed variations in morphology (reduced chain length) and metabolic profile (lipid and nitrogen metabolism, cell cycle regulation, and frustule formation).

**TABLE 1:** Summary of the current literature reports on the characterization of infochemicals.

**Producer**	**Receiver**	**Properties**	**References**
*Daphnia* spp.	*Scenedesmus* sp.	<0.5 kDa;	Lampert et al., 1994
		Insensitive to proteases;	
		Heat and pH stable;	
		Non-volatile;	
		Sensitive to incineration	
*Daphnia* spp.	*Scenedesmus* sp.	Lipophilicity increased at low pH;	von Elert and Franck, 1999
		Olefinic double bonds;	
		Insensitive to sulphatase, phosphatase and proteases;	
		Not free fatty acids	
*Daphnia* spp.	*Scenedesmus* sp.	Non-volatile	van Holthoon et al., 2003
*Daphnia* spp.	*Actinastrum* sp.	Not butanoic acid, acetic acid or amino acids	Yasumoto et al., 2005
*Daphnia* spp. (homogenates)	*Scenedesmus* sp.	Aliphatic Sulfates and Sulfamates	Yasumoto et al., 2005, 2008
*Daphnia* spp.	*Scenedesmus* sp.	Anionic Surfactants	Yasumoto et al., 2005
*Daphnia* spp.	Green algae	8-methylnonilsulfate	Uchida et al., 2008
		Sulfates	
		Amidosulfates	

Although transcriptomics provides a global overview of the potential response to the chemical cues, it only enables prediction of the metabolic responses, as transcripts do not perform actual function themselves and it fails to incorporate post-transcriptional control mechanisms. A more accurate physiological understanding can be provided by quantifying protein expression, as proteins perform functional roles in cells. However, to our knowledge, no proteomics experiments have been performed to decipher algal responses to grazer cues. Some proteomics analyses have been carried out to investigate defensive responses in microalgae. For example, *Chlamydomonas reinhardtii* produce palmelloids (aggregates of cells in a multicellular complex, separated from one another but embedded in a mucilaginous material; Bausor and Agona, 1973) as a defense response against increased salinity levels. Khona et al. (2016) investigated the variations in the proteome of the *Chlamydomonas reinhardtii* under salt stress. Cellular changes included mechanisms for starch and lipid accumulation, the production of an extracellular polysaccharide envelope, and a cell cycle mechanism arrest which implicated the involvement of cell wall proteins such as expansin, WSC (Wall Stress-responsive Component) domain protein, pheophorin-C5, VSP4 (Vacuolar Sorting Protein 4), and Cathepsin-Z-like proteins.

In this study, we applied iTRAQ for a quantitative assessment of the proteomic response of the freshwater microalga *Scenedesmus subspicatus* to naturally occurring infochemicals from the grazer *Daphnia magna*. As the fully annotated genome of *S. subspicatus* is not available, we compiled a database of known protein sequences from all annotated and genome sequenced green microalgae and cyanobacteria, relying on shared peptide homologs for identification (Pandhal et al., 2009a, b). Our experiment was designed to compare between infochemicals treated and untreated *S. subspicatus* cells, as well as compare *S. subspicatus* cells in flocs (where unicells are grouped in clusters and held together by an extracellular “sticky” matrix), versus planktonic cells, dominated by *coenobia*. Our objective was to reveal major metabolic pathways altered by exposure to the *Daphnia* cues, providing a better understanding of colony formation and aggregation processes in *S. subspicatus.* Proteomics provides a unique profile of metabolic function and therefore our work targets specifically the molecular mechanisms taking place. From an ecological and evolutionary perspective, this information represents a step toward a more comprehensive understanding of the effect of infochemicals on species interactions and how they affect the structure of aquatic communities. From an engineering and biotechnological perspective, this information represents a decisive advance into exploiting a low cost and sustainable algal biomass harvesting method without the requirement for chemical flocculants.

## Materials and Methods

Unless otherwise stated, all chemicals were supplied by Sigma-Aldrich (Poole, Dorset, United Kingdom) with the highest purity available. All solvents were high-performance liquid chromatography (HPLC) grade and supplied by Thermo Fischer Scientific (Paisley, United Kingdom).

### Algal Cultivation and Infochemicals Production

*Scenedesmus subspicatus*, strain NIVA-CHL 97 (RRID:SCR_011473) was maintained in Ebert’s medium (Ebert, 2013) and cultured in 250 mL Erlenmeyer flasks at 20 ± 1°C, continuously illuminated under light at 259 μmol m^–2^ s^–1^ until exponential phase was reached at ∼2⋅10^6^ cells mL^–1^. *Daphnia magna* used to produce the infochemicals was a laboratory clone (RRID:SCR_008148) maintained in the lab for several months in a temperature-controlled room at 20 ± 1°C in a 16:8 h light-dark cycle, cultured in 1 L jars in ASTM hard water (ASTM, 1980) and fed daily with 250 μL of *S.* subspicatus cells (2⋅10^5^ cells mL^–1^). To produce the infochemicals, *D. magna* was incubated at a density of 100 ind L^–1^ with *S. subspicatus* as food for 24 h. The culture was then filtered through a 0.2 μm cellulose acetate filter (Sartorius Stedim Biotech Gmbh, Germany) to obtain the *Daphnia* infochemicals containing water (DapW).

### Experimental Design

**FIGURE 1:**
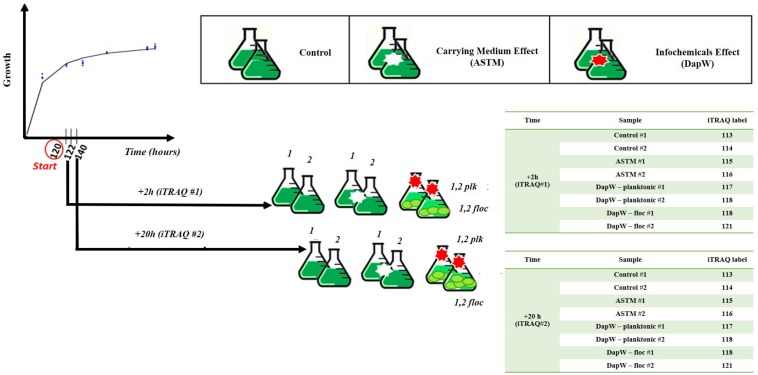
Experimental design scheme.

Five mL of exponentially growing *S. subspicatus* (∼2⋅10^6^ cells mL^–1^) was transferred to 250 mL Erlenmeyer flasks containing 150 mL of sterile Ebert’s medium and allowed to grow until early exponential stage (∼2⋅10^6^ cells mL^–1^), as established by growth experiments. At this point, either 5 mL of additional culture medium (control) or five mL of either DapW or ASTM water were added to the biological replicates (*n* = 3). Batch cultures were incubated at 20 ± 1°C on a shaking table at 120 rpm, continuously illuminated under light at 259 μmol m−^2^ s^–1^ and randomly rearranged daily. Sampling was performed after 2 h (+*2 h*) and 20 h (+*20 h*) of exposure. These time points were chosen to observe early variations under infochemicals effects and at a time after which no further flocculation is observed. At the selected time points, from each biological replicate of Control and +ASTM cultures we collected 150 ml aliquots. For *S. subspicatus* cultures treated with infochemicals, two fractions were collected: 50 ml aliquots for the lower part- *flocs*, dominated by unicells, and 100 ml aliquots for the upper part – *planktonic cells*, mostly composed by *coenobia*, as confirmed by composition studies. A schematic representation of the experimental design is reported in Figure 1.

### Composition, Growth and Flocculation Efficiency

One mL aliquots of algal cultures were sampled on alternate days and fixed in Lugol’s dilute solution. Growth rates and culture composition were determined by cell counting and flow cytometry, using a hemocytometer (Neubauer Improved Superior, Germany) under a microscope (Kyowa, Medilux-12), and a Beckman Coulter CytoFLEX S, respectively. Cell counting data were reported as percentage distributions of unicells, 2-, 3-, 4-, 8- celled *coenobia*, respectively, whilst flow cytometry data were reported as a function of the median SSC-H (Side Scattering-Height) (*n* = 3) (Bakke, 2001; Marie et al., 2005; Peniuk et al., 2016). For cells exposed to infochemicals, we separately analyzed the upper *planktonic* fraction (DapW _*plk*_) and the lower *floc* fraction (DapW _*floc*_). Analysis of flocs was done by initial mechanical disaggregation (i.e., manually agitating the growth flasks), followed by counting of the constitutive cells using the flow cytometer described above. SSC-Hs were extrapolated using FlowJo software (RRID:SCR_008520), and the data was analyzed by ANOVA. Macro and microscopic pictures of *S. subspicatus* cultures flocculated under the effect of infochemicals were taken using a Samsung-Galaxy A5 phone-integrated camera as well as a microscope with 400× magnification (Leitz Wetzlar, Germany) embedded with a camera (QIMAGING, MicroPublisher 3.3 RTV) and connected to a computer with the software QCapturePro Version 5.1.1.14 (RRID:SCR_014432) respectively. Flocculation efficiency was determined by measuring the optical density (OD) of cultures at 680 nm before adding infochemicals and the residual OD of the supernatant after 20 h exposure. OD readings were taken using a UV/Vis spectrophotometer (UltroSpec 3000, Pharmacia Biotech, Biochrom Ltd. Cambridge, United Kingdom) at 680 nm and flocculation efficiency calculated using Equation 1.

(1) $$\text{Flocculation Efficiency FE}\left(\%\right)=\frac{\left(O\,D_{t\,0}-O\,D_{t}\right)}{O\,D_{t\,0}}.100$$

Differences in flocculation efficiencies were examined by ANOVA and *post hoc* Tukey test.

### Proteomics

#### Protein Preparation and Quantification

Among the available techniques for quantitative proteomics, we chose iTRAQ in this study, as it is a well-established chemical labeling method in quantitative proteomics (Evans et al., 2012; Couto et al., 2014; Helliwell et al., 2017; Shi et al., 2017; Flores et al., 2019). As schematically reported in Figure 1, Control, +ASTM and +Infochemicals algal cultures were harvested after five days of growth plus either +*2 h* (*n* = 2) or +*20 h* (*n* = 2) of exposure, and then pelleted them by centrifugation at 3000 × *g* for 15 min at 4°C. Cultures exposed to infochemicals exhibited flocculation, therefore we separated the supernatant (planktonic) fraction from the floc fraction. Algal cell pellets were resuspended in 200 mM triethylammonium bicarbonate buffer (TEAB), pH = 8.0 and transferred to a protein Lo-bind tube and centrifuged again at 3000 × *g* for 10 min. The cells pellets were resuspended in 250 μL of lysis buffer composed of 200 mM TEAB, 10 mM DL-dithiothreitol (DTT), 0.5% (w/v) sodium deoxycholate and 1 μL per mL enzymatic plant protease inhibitor. Cells were lysed by liquid nitrogen cracking and subsequent bead-beating. Unbroken cells and cell debris were pelleted by centrifugation at 18000 × *g* for 5 min and the supernatants transferred to clean Lo-Bind tubes. We estimated the total protein concentration by the modified Lowry method (Flores et al., 2019).

#### Proteins Digestion and Labeling

One hundred micrograms of protein extracted from the control, ASTM-treated, infochemicals-exposed planktonic cells and infochemicals-exposed flocs, at +*2 h* and +*20 h*, were subjected to reduction, alkylation, digestion using trypsin and labeling with iTRAQ reporters, as described below. Reduction was performed using 10 mM Tris-(2-carboxyethyl)-phosphine (TCEP) final concentration, followed by incubation of samples at 60°C for 30 min. Alkylation was performed using 20 mM final concentration of methyl methanethiosulfonate (MMTS) and samples were incubated for 30 min at room temperature. Samples were then digested with 1:20 sequencing grade modified trypsin (Promega Corporation, United States) in 200 mM TEAB and incubated overnight at 37°C. Two sets of iTRAQ 8-plex labels were used for this study as described in Figure 1, one set for +*2 h* exposure and the other set for +*20 h* exposure. iTRAQ labeling was performed following the manufacturer’s instructions (AB Sciex, United States). The labeled peptides were combined in one tube and dried overnight in a vacuum centrifuge at 30°C.

#### HPLC Fractionation

Dried iTRAQ-labeled peptides were resuspended in buffer A [3% (v/v) acetonitrile and 0.1% (v/v) trifluroacetic acid (TFA) in HPLC water] and off line fractionated using an Hypercarb Porous Graphite column (Thermo Fisher Scientific, United Kingdom), with 3 μm particle size, 50 mm length, 2.1 mm diameter and 250 Å pore size. Peptides were reverse-phase separated using buffer A [3% (v/v) acetonitrile and 0.1% TFA in water] and buffer B [97% (v/v) acetonitrile and 0.1% TFA in water]. The Hypercarb separation was performed on a Dionex UltiMate 3000 Autosampler linked to Dionex UltiMate 3000 Flow Manager and Pump system (Thermo Scientific, United Kingdom). Gradient elution was performed at a flow rate of 30 μL min^–1^ as follows: 3% B -10% B for 10 min, 10% B – 50% B for 75 min, 50% B – 90% B for 1 min, 90% B for 10 min, 3% B for 14 min. The fractions were collected every 2 min from 10 to 120 min, and dried by vacuum centrifugation (Scanvac Labogene, Denmark) ready for reverse-phase LC-MS/MS.

#### LC MS/MS

Samples were resuspended in 10 μL buffer A [3% (v/v) acetonitrile and 0.1% (v/v) formic acid (FA) in HPLC water] before loading onto an Easy-Spray C18 column (75 μm × 50 cm) at a flow rate of at 300 nL min^–1^ with a 2-step gradient from 97% buffer A (0.1% (v/v) FA in HPLC water) to 4% Buffer B (80% (v/v) acetonitrile and 0.1% (v/v) FA in HPLC water) over 5 min, then 4–40% buffer B over 100 min, then 40–90% buffer B for 1 min, 90% buffer B for 14 min, 90% to 4% buffer B for 1 min and 4% buffer B for 14 min. Mass spectrometry was performed using a hybrid quadrupole-orbitrap mass spectrometer (Q Exactive HF, Thermo Scientific) connected to an UPLC U3000 RSLC nano (Thermo Scientific, United Kingdom). Mass spectrometry (MS) data was acquired using Xcalibur software v 4.0 (RRID:SCR_014593) with the following settings. MS scans were acquired with 60,000 resolution, automatic gain control (AGC) target 3e6, maximum injection time (IT) 100 ms. The MS mass range was set to be in the range 100–1500 m/z. Tandem mass spectrometry (MS/MS) scans were acquired using high-energy collision dissociation (HCD), 30,000 resolution, AGC target 5e4, maximum IT 120 ms. In total, 15 MS/MS were acquired per MS scan using normalized collision energy (NCE) of 34% and isolation window of 1.2 m/z.

#### Data Analysis

Raw data was processed using MaxQuant, Version 625 1.5.4.1 (RRID:SCR_014485) (Michalski et al., 2011). The settings were as follows. For “type of experiment” MS2 and 8-plex iTRAQ were selected with reporter mass tolerant 0.01 Da. Enzymatic digestion with trypsin was specified and two missed cleavages were allowed per peptide. Oxidation of methionine and deamidation of asparagine and glutamine were selected as variable modification and methylthio modification of cysteine was selected as the fixed modification. The false discovery rate (FDR) at the peptide spectrum match/protein level was set at 1%. Raw data was interrogated against a.fasta file which was generated using proteomes from green microalgae and cyanobacteria data with a total of 97,523 entries (downloaded from UniProt on June 2017). Isotopic and median corrections were applied using an in-house automated method as described previously (Ow et al., 2009, 2010; Noirel et al., 2011), using the following settings: false discovery rate (FDR) = 1%; required unique peptides = ≥2, *t*-test threshold = 0.05, multiple test correction = off. A summary of the phenotype comparisons and their supporting biological motivations is reported in Table 2. Fold changes of the differentially regulated proteins were calculated using a method described by Pham et al. (2010), with 95% significance. Since two biological replicates were available for each condition, a change was only reported if it was significant in both i.e., all four *p*-values must have at least 95% significance (Pham et al., 2010). The mass spectrometry proteomics data have been deposited to the ProteomeXchange Consortium via the PRIDE (RRID:SCR_004055) (Perez-Riverol et al., 2018) partner repository with the dataset identifier PXD014153. We applied Principal Components Analysis (PCA) to the isotope and median corrected reporter ions intensities (113, 114, 115, 116, 117, 118, 119, 121) to first check on biological groupings (*n* = 2) and second to formally test whether the treatments were significantly different with respect to the PCA axes, using a permutation-based analysis of variance (Adonis method), using the *rda* and *adonis* functions from the R package Vegan (Oksanen, 2015) (RRID:SCR_011950). The major axes returned by the PCA also offered a first insight into proteins linked, via abundance, to different treatments. We used VENN diagrams (BioVenn, Hulsen et al., 2008) to identify shared and differentially expressed proteins (DEPs) among and between control and treatments, using their relative fold change abundance variations. The resulting unique DEPs were functionally classified using the KO (KEGG Orthology) and BRITE functional hierarchies obtained using the KAAS - KEGG Automatic Annotation Server (RRID:SCR_001120). We used the following settings: Search program: BLAST; Query sequences (in multi-FASTA): Text data (downloaded from UniProt on June 2017); GENES data set: manual selection → organisms list → selected organisms: Green algae, Amborella family: *Chlamydomonas reinhardtii; Ostreococcus lucimarinus; Ostreococcus tauri, and Micromonas commoda*”; Assignment methods: BH (bi-directional best hit). KAAS results contained KO (KEGG Orthology) assignments and automatically generated KEGG pathways. KO assignments were based on the best hit information using Smith-Waterman scores as well as by the manual curation. Each K number represented an ortholog group of genes, and it was directly linked to an object in the KEGG pathway map or the BRITE functional hierarchy. We based the hierarchical clustering of the unique DEPs on the fold change expression values and we implemented them in R using the package *pheatmap* (RRID:SCR_003005).

**TABLE 2:** Phenotypes comparisons and related biological motivations.

**Time Point**	**Phenotypes comparison**	**Biological Motivations**	
+2h	ASTM vs. Control	Changes due to the presence of salts in the *Daphnia* culturing medium – “carrier effect”	*Changes caused at the alarm phase -upon early detection of cues*
	DapW_*plk*_ vs. Control/ASTM	Changes due to infochemicals – colony formation	
	DapW_*floc*_ vs. Control/ASTM	Changes due to infochemicals - flocculation	
	DapW_*floc*_ vs. DapW_*plk*_	Colony formation vs. flocculation	
+20h	ASTM vs. Control	Changes due to the presence of salts in the *Daphnia* culturing medium – “carrier effect”	*Changes caused at the acclimation phase – after which no increase of*
	DapW_*plk*_ vs. Control/ASTM	Changes due to infochemicals – colony formation	*flocculation efficiency is observed*
	DapW_*floc*_ vs. Control/ASTM	Changes due to infochemicals - flocculation	
	DapW_*floc*_ vs. DapW_*plk*_	Colony formation vs. flocculation	

### Extraction and Analysis of Soluble EPS (sEPS)

Hundred mL aliquots from Control, +ASTM and +Infochemicals algal cultures were centrifuged at 4500 × *g* for 15 min at 4°C to extract sEPS. Supernatant was first passed through a 0.22 μm pore-size filter and dialyzed against distilled water using a SnakeSkin Dialysis Tubing (3.5 kDa MWCO, Thermo Scientific). After dialysis the sEPS were freeze-dried (CoolSafe, ScanVac, LaboGene) and re-suspended in 1200 μl of HPLC grade water for further quantification assays. Carbohydrates were measured using the anthrone method using glucose as a reference standard (Le and Stuckey, 2016). Proteins were measured using the BCA assay kit (QuantiPro^TM^ BCA Assay Kit, Sigma Aldrich) using bovine serum albumin (BSA) as a reference standard (Georgiou et al., 2008). Fatty acids were measured using the method reported by Kapoore (2014). The concentrations of each fraction were normalized by the algal cells’ concentration.

### Intracellular Carbohydrates and Fatty Acids Analysis

For carbohydrates analysis, 5 mL aliquots from control and treatment cultures (*n* = 3) were centrifuged at 4500 × *g* or 10 min and pellets stored at −20°C until analysis. Concentration of intracellular carbohydrates was estimated using the anthrone method with glucose as a reference standard (Le and Stuckey, 2016). For fatty acids analysis, 5 mL biomass aliquots from control and treatment cultures (*n* = 3) were pelleted by centrifugation at 19000 × *g* for 3 min to which 1.2 mL of a 1:2 methanol:chloroform (v/v) mixture and an equal volume of glass beads (425–600 μm, acid washed) were added. Cells were disrupted with a Genie cell disruptor (Scientific Industries Inc., NY, United States) for 15 cycles (1-min bead beating and 1-min stand in an ice bath). After cell disruption, the supernatant was collected after centrifugation at 19000 × *g* at 4°C for 10 min and added to 800 μL of 1:1 chloroform and water (v/v). After further centrifugation at 8000 *g* at 4°C for 10 min the organic phase was pre-weighed prior to evaporation under inert nitrogen gas using a six-port mini-vap evaporator (Sigma-Aldrich, Dorset, United Kingdom) and stored at −80°C until further analysis. The extracted lipids were converted into fatty acid methyl esters (FAMEs), as described by Kapoore (2014) with minor modifications: 250 μL of 1:1 chloroform:methanol (v/v) and 100 μL of 10% (w/v) boron trifluoride:methanol was added to the dried extract and incubated at 80°C for 90 min. Afterward, the samples were added to 300 μL water and 600 μL hexane and centrifuged at 18000 × *g* at 4°C for 10 min. Five hundred microliters of the organic phase was then removed and evaporated to dryness under inert nitrogen gas. The dried FAMEs were reconstituted in 100 μL hexane prior to identification and quantification on a TRACE 1300 gas chromatography flame ionization detector (GC-FID) System (Thermo Scientific, Hertfordshire, United Kingdom) using a TR-FAME capillary column (25 m × 0.32 mm × 0.25 μm). One microliter derivatized sample was injected in split injection mode at 250°C (split flow 75 mL min^–1^ and purge flow 5 mL min^–1^). The GC-FID was operated at a constant flow of 1.5 mL min^–1^ helium at an initial temperature of 150°C for 1 min, followed by ramping at 10°C min^–1^ to 250°C and held constant here for 1 min. Peak identities were ascertained using an external standard 37 component FAME mix (Supelco, United States) and peak areas were integrated using a chromatography data system (Thermo Scientific Dionex Chromeleon^TM^ 7 software, Version 7.2.0.4154) (RRID:SCR_016874). In total, five technical replicates were run, among which only the FAMEs identified in three or more replicates were considered true hits.

## Results

### Growth, Composition and Flocculation Efficiency

**FIGURE 2:**
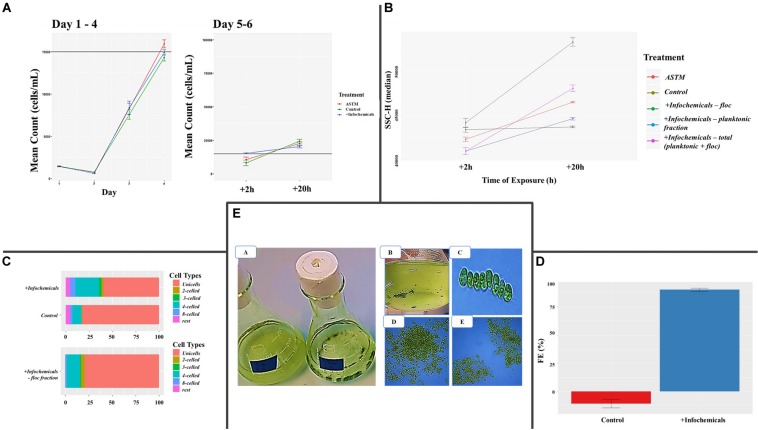
**(A)** Algal growth curves [before and after addition of either additional medium (Control), ASTM or infochemicals], with details on +*2 h* and +*20 h* exposure. Data are reported as (cells ⋅ mL^–1^) ± SE per day (*n* = 3). **(B)** Flow Cytometry Side SCatter pulse Height (SSC-H) median values, used to differentiate algal phenotypes and correlate them with cells size (*n* = 3). **(C)** Percentage distribution of unicells and *coenobia* in *S. subspicatus* cells exposed to infochemicals, compared to control (*n* = 3). **(D)** Flocculation Efficiency (%) ± SE (*n* = 3). **(E)** Macro and microscopic pictures of infochemicals induced flocs (A, B, D, E) and *coenobia* (C).

Growth rate was not affected by addition of either ASTM or infochemicals, compared to Control (Figure 2A). At +*2 h*, we did not detect significant SSC-H differences between *S. subspicatus* cells exposed to ASTM or Control. Also, planktonic and floc fractions of algae exposed to infochemicals (DapW _*plk*_ and DapW _*floc*_) did not show significantly different SSC-H median values from each other as well as from ASTM and Control (Figure 2B). At +*20 h*, *S. subspicatus* cells exposed to ASTM were significantly different from Control (*p* < 0.001). Also, DapW _*floc*_ showed significant SSC-H variations from both DapW _*plk*_ as well as ASTM and Control (*p* < 0.001). However, the SSC-H for DapW _*plk*_ was significantly different from Control (*p* = 0.330) but not from ASTM (*p* = 0.028) (Figure 2B). The observed trends for SSC – H median values were correlated to the relative distribution of unicells and *coenobia*. Specifically, at +*20 h* we noticed a significant increase in the mean number of *coenobia* in the cultures of *S. subspicatus* exposed to infochemicals, whereas the Control cultures were dominated by unicells (>70%) (Figure 2C). A further analysis of the floc fraction of *S. subspicatus* cells exposed to infochemicals, showed that they did not consist of *coenobia*, being instead predominantly composed of unicells (Figure 2C). Flocculation efficiency (FE) differed significantly between Control, where no flocculation occurred, and *S. subspicatus* exposed to infochemicals (*p* = 0.0009), with FE = 77.37 ± 16.93% (Figure 2D). In Figure 2E, we reported the macro and microscopic pictures of infochemicals induced flocs and *coenobia*.

### Proteomics Analyses

#### Overview of Proteomics

**FIGURE 3:**
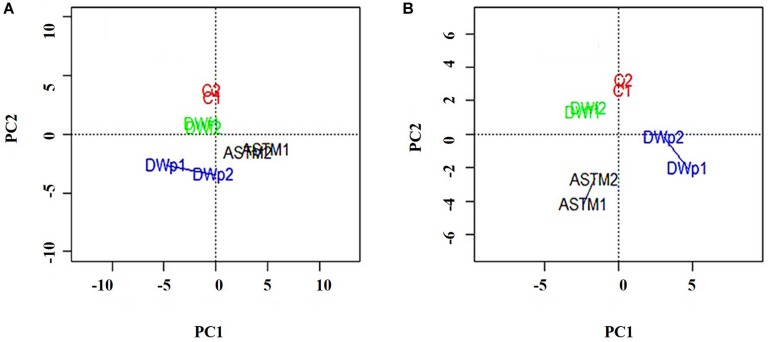
PCA plots of the 8 samples, clustered by biological replicates (*n* = 2). Clusters show Control conditions (red), ASTM addition conditions (black), addition of infochemicals – planktonic fraction (blue) and addition of infochemicals – floc fraction (green). **(A)** +*2 h*; **(B)** *20 h.*

**FIGURE 4:**
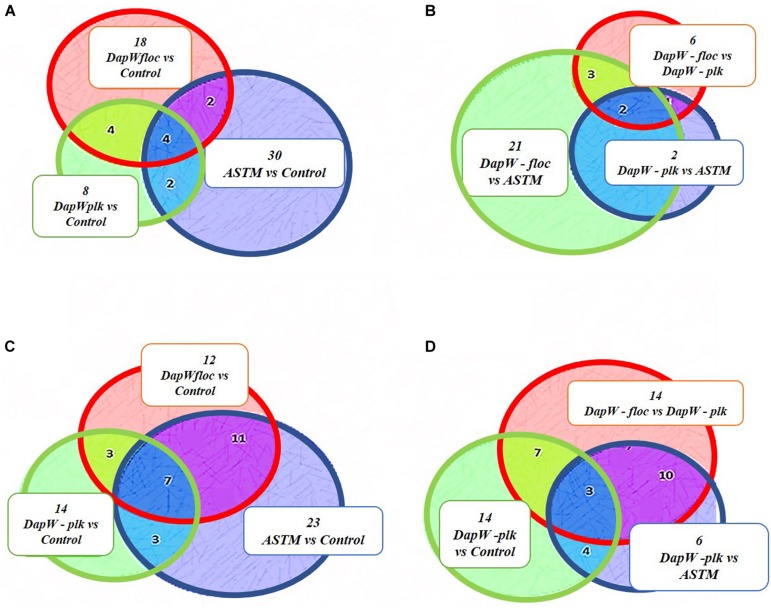
Venn Diagrams of DEPs. **(A,B)** +2 h; **(C,D)** +20 h.

A total of 46,720 MS/MS scans were registered and identified 465 protein groups for the +*2 h* time-point, while 47,346 MS/MS and 452 protein groups were obtained for the +*20 h* time-point. As the *S. subspicatus* genome is not fully sequenced it was necessary to rely on matching experimental spectra to peptides from a library of closely related organisms. The most referenced proteomes observed belonged to *Tetradesmus obliquus* (previously reported as *Scenedesmus obliquus*) (9% of total peptides matched), *Chlamydomonas reinhardtii* (8%), *Volvox carteri f. nagariensis* (8%), *Coccomyxa subellipsoidea* (8%), *Scenedesmus armatus* (7%), *Chlorella variabilis* (7%), *Dunaliella salina* (6%), *Scenedesmus bijugus* (5%), *Dunaliella tertiolecta* (4%), *Bathycoccus prasinos* (4%), *Microcystis aeruginosa* (4%), *Scenedesmus quadricauda* (3%), *Cyanophora paradoxa*, (3%), *Ectocarpus silicosus* (3%), *Ostreococcus luciminarus* (3%), *Micromonas pusilla* (2.5%), and *Scenedesmus acutus* (1.5%). Figure 3 shows the PCA clustering at both time-points (+*2 h* and +*20 h)*, indicating how in both cases different treatments were clearly separated. This suggested that protein abundance changed upon exposure to infochemicals and with close grouping between the biological replicates, indicating that the biological replicates were similar enough to allow meaningful insights from the comparison of phenotypes between groups. Permutation-based analysis of variance confirmed treatments were significantly different from the control (number of permutations = 999, +*2 h*-*p_*val*_* = 0.005, +*20 h*-*p_*val*_* = 0.007). The first principal component (dimension 1) accounts for as much variation in the dataset as possible (+*2h* PC1: 71%, +*20 h* PC1: 47.5%); therefore the top 1% contributors to PCA-dimension 1 are reported in Tables 3, 4, with the identification of the biological process involved to provide a better description of how the biological treatments are differentiated. The Venn diagrams of the DEPs are presented in Figures 4A,B for +*2 h* and Figures 4C,D for +*20 h*. The sum of the numbers in each large circle presents the total number of DEPs among various combinations while the overlapping parts of the circles show common DEPs between combinations (Table 5).

**TABLE 3:** Top 1% PCA contributors to dimension 1.

**Entry**	**Protein names**	**Organism**	**Gene ontology (biological process)**
*E1ZJQ8*	NADH dehydrogenase [ubiquinone] flavoprotein 1, mitochondrial (EC 1.6.5.3) (EC 1.6.99.3) (Fragment)	*Chlorella variabilis* (Green alga)	Electron transport, respiratory chain
*D8U1R3*	Uncharacterized protein	*Volvox carteri f. nagariensis*	Protein metabolic process [GO:0019538]
*E1ZFQ1*	Uncharacterized protein	*Chlorella variabilis* (Green alga)	Metabolic process [GO:0008152]
*I0YV40*	Cofactor-independent phosphoglycerate mutase	*Coccomyxa subellipsoidea* (strain C-169) (Green microalga)	Glucose catabolic process [GO:0006007]
*I0YL77*	ADP-ribosylation factor 1	*Coccomyxa subellipsoidea* (strain C-169) (Green microalga)	Small GTPase mediated signal transduction [GO:0007264]
*D8TIF4*	Uncharacterized protein	*Volvox carteri f. nagariensis*	
*E1ZQ02*	Uncharacterized protein	*Chlorella variabilis* (Green alga)	Proteolysis [GO:0006508]

*+*2 h* exposure.*

**TABLE 4:** Top 1% PCA contributors to dimension 1.

**Entry**	**Protein names**	**Organism**	**Gene ontology (biological process)**
*D8U1I3*	Adenylosuccinate synthetase, chloroplastic (AMPSase) (AdSS) (EC 6.3.4.4) (IMP–aspartate ligase)	*Volvox carteri f. nagariensis*	’*De novo*’ AMP biosynthetic process [GO:0044208]
*D8U4Q1*	Uncharacterized protein	*Volvox carteri f. nagariensis*	Metabolic process [GO:0008152]
*E1ZD58*	Cysteine synthase (EC 2.5.1.47) (Fragment)	*Chlorella variabilis* (Green alga)	Cysteine biosynthetic process from serine [GO:0006535]
*A8IW00*	Glutamine synthetase (EC 6.3.1.2)	*Chlamydomonas reinhardtii* (*Chlamydomonas smithii*)	Glutamine biosynthetic process [GO:0006542]
*D8TKE8*	Mg-protoporphyrin IX chelatase (EC 6.6.1.1)	*Volvox carteri f. nagariensis*	Chlorophyll biosynthetic process [GO:0015995]; photosynthesis [GO:0015979]
*E1Z349*	Malate dehydrogenase (EC 1.1.1.37)	*Chlorella variabilis* (Green alga)	Carbohydrate metabolic process [GO:0005975]; malate metabolic process [GO:0006108]; tricarboxylic acid cycle [GO:0006099]
*K8EQC7*	Uncharacterized protein	*Bathycoccus prasinos*	

*+*20 h* exposure.*

**TABLE 5:** Number of unique DEPs for each phenotype comparison and at +*2 h*, +*20 h* exposures.

**Time point**	**Phenotypes comparison**	**Unique DEPs**
+*2 h*	DapW_*floc*_ vs. Control	18
	DapW_*plk*_ vs. Control	21
	ASTM vs. Control	30
	DapW_*floc*_ vs. DapW _*plk*_	6
	DapW_*floc*_ vs. ASTM	21
	DapW_*plk*_ vs. ASTM	2
+*20 h*	DapW_*floc*_ vs. Control	12
	DapW_*plk*_ vs. Control	14
	ASTM vs. Control	23
	DapW_*floc*_ vs. DapW _*plk*_	14
	DapW_*floc*_ vs. ASTM	14
	DapW_*plk*_ vs. ASTM	6

#### Proteomics Patterns

**FIGURE 5:**
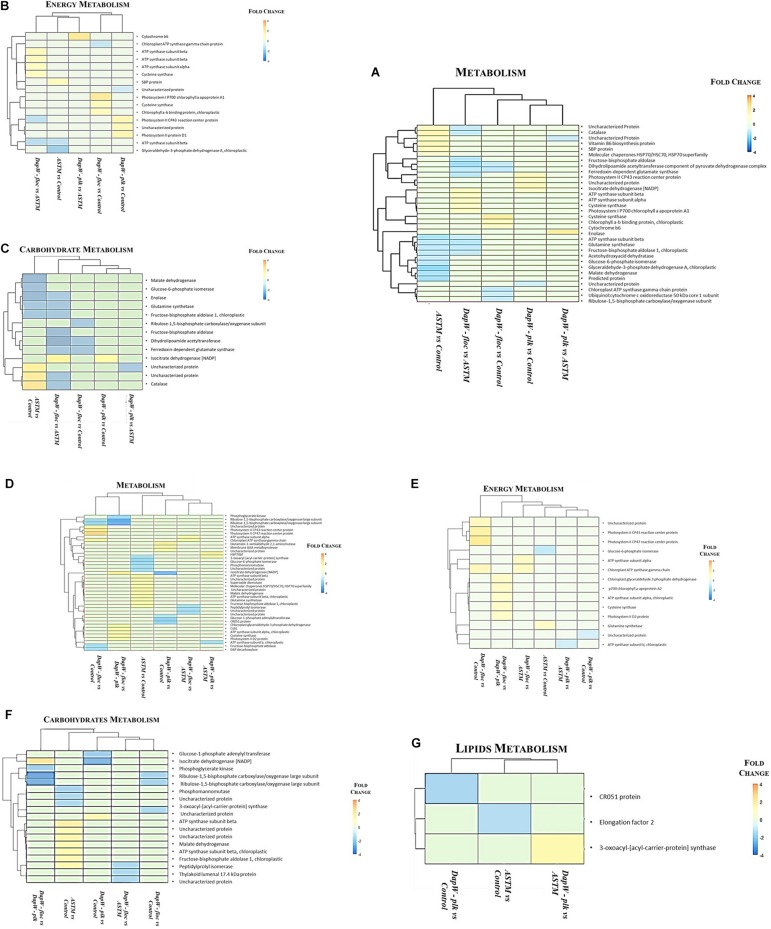
Hierarchical clustering of unique DEPs with similar functions under infochemicals exposure. **(A–C)** +*2 h*; **(D–G)** +*20 h* exposure.

We classified unique DEPs according to their biological functions into the following main categories: Energy, Carbohydrates and Lipids metabolism (Figure 5). At +*2 h* (Figures 5A–C) hierarchical clustering of DEPs for energy metabolism, inclusive of photosynthesis, sulfur metabolism, carbon fixation in photosynthetic organisms, and oxidative phosphorylation showed two main clusters: (1) Infochemicals exposed algal cells-floc fraction (DapW _*floc*_) against ASTM exposed cells and (2) both Planktonic fraction (DapW _*plk*_) and floc fraction (DapW _*floc*_) of *S. subspicatus* exposed to infochemicals, against Control. For both clusters, unique DEPs showed higher abundance. For carbohydrate metabolism [which included glyoxylate and dicarboxylate metabolism, glycolysis/gluconeogenesis, citrate cycle (TCA), and the pentose phosphate pathway], it was shown how the proteomes of DapW _*plk*_ against either ASTM or Control were clustered together, as it was for DapW _*floc*_ fraction against both ASTM and Control. After +*20 h* exposure (Figures 5D–G) and for energy metabolism, unique DEPs related to DapW _*floc*_ against either Control or ASTM or DapW _*plk*_ were more abundant, while unique DEPs linked to DapW _*plk*_ were less abundant when compared against both Control’s and ASTM’s unique DEPs. For carbohydrates metabolism, which accounted for glycolysis/gluconeogenesis, glyoxylate and dicarboxylate metabolism, glycolysis/gluconeogenesis, citrate cycle (TCA), pentose phosphate pathway and starch and sucrose metabolism, unique DEPs were less abundant for both DapW _*plk*_ and DapW _*floc*_ compared to Control as well as ASTM. At this time of exposure, we could observe the additional category of lipids metabolism, in particular, the biosynthesis of fatty acids, which showed a higher abundance of DEPs associated to DapW _*plk*_.

### sEPS

**FIGURE 6:**
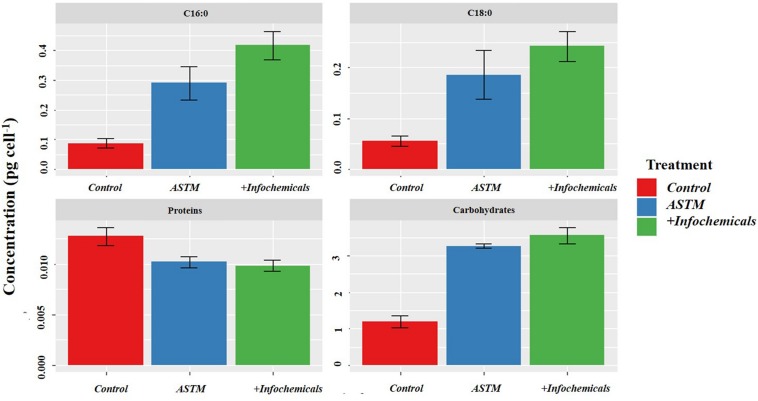
sEPS in *S. subspicatus* after exposure to *Daphnia* infochemicals, compared to control cultures. Results are reported as mean ± SE (*n* = 3).

Figure 6 shows variation in carbohydrate, protein, and fatty acid contents in sEPS relating to +*20 h* time of exposure of *S. subspicatus* to infochemicals, compared to Control (no infochemicals) and cultures exposed to the ASTM. Overall, we observed a general increase in carbohydrates content and a decrease of total protein concentration in both ASTM and infochemicals exposed *S. subspicatus* cells, compared to Control, although not statistically significant. Interestingly, we detected an overall increase in the amount of palmitic and stearic acids (C16:0 and C18:0, as FAMEs) for algal cells exposed to both infochemicals and ASTM, compared to control cultures.

### Intracellular Carbohydrates and Fatty Acids Analysis

**FIGURE 7:**
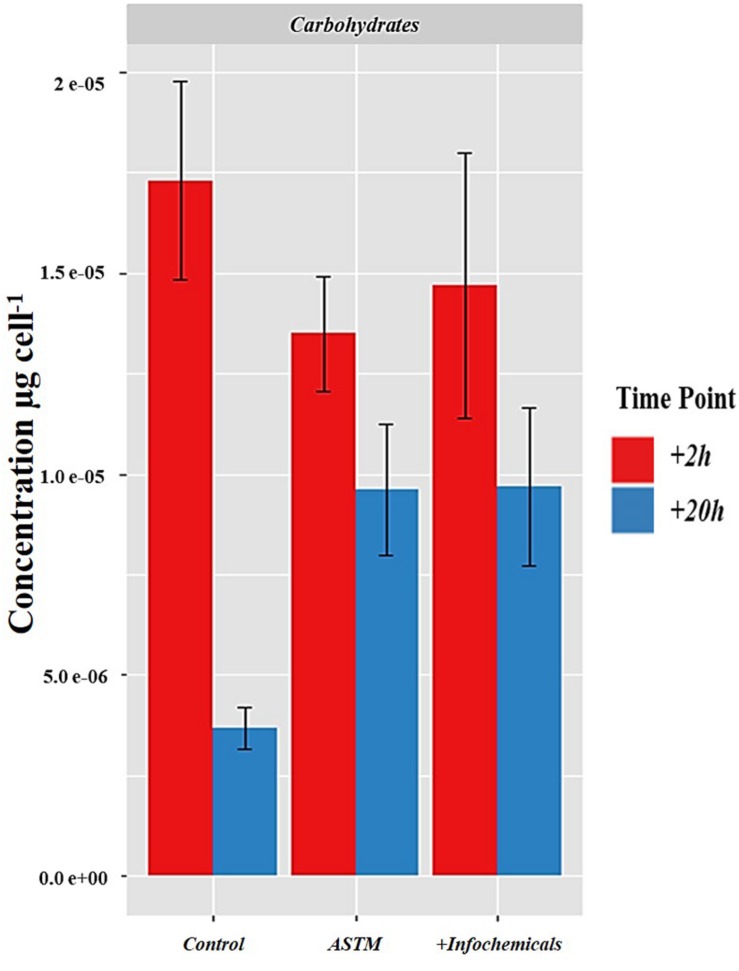
Intracellular variation in carbohydrate concentration of *S. subspicatus* cells, after exposure to either *Daphnia* infochemicals or ASTM as compared to control cultures. Results are reported as mean ± SE (*n* = 3).

**FIGURE 8:**
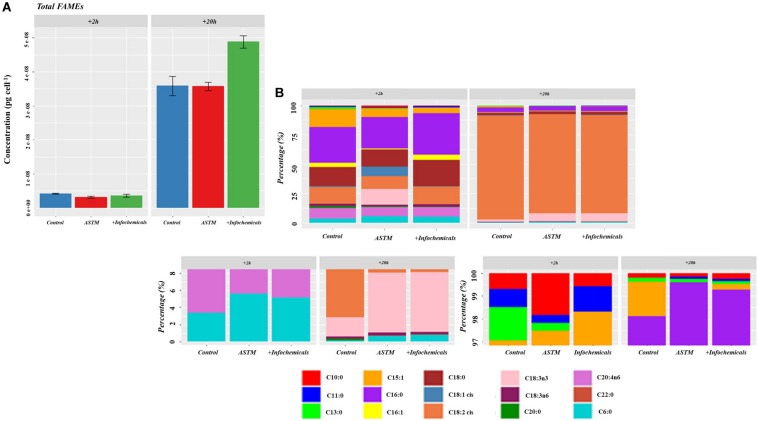
**(A)** Total amount of intracellular fatty acids (as FAMEs). Results are reported as mean of the concentration (μg ⋅ cells^– 1^) ± SE (*n* = 3). **(B)** Relative fatty acids (as FAMEs) distribution (Top Panel: overall distribution; Lower Panels: zoomed areas) after exposure to either ASTM or *Daphnia* infochemicals at +*20 h*.

Figure 7 shows the intracellular variation in carbohydrate concentration of *S. subspicatus* cells. Results indicated an overall trend toward decrease of carbohydrates from +*2 h* to +*20 h* exposure to infochemicals, although this was not significantly different. We also evaluated quantification and distribution of intracellular FAMEs in *S. subspicatus* cells exposed to infochemicals (Figure 8A). Results showed that at +*2 h* there were no significant differences in total amounts of intracellular FAMEs among treatments and control. However, at +*20 h* the total fatty acids content almost doubled for algal cells exposed to infochemicals. Interestingly, we observed different distributions of individual FAMEs at both times of exposure (Figure 8B). Specifically, we detected an increase of saturated and unsaturated FAMEs for *S. subspicatus* exposed to infochemicals, and the presence of n-3 polyunsaturated FAMEs (PUFAs) for algae exposed to ASTM only. At +*20 h* we observed an accumulation of n-2 PUFA FAMEs for all treatments and control. Also, compared to both ASTM and Control cultures, we observed that the relative proportions of unsaturated and saturated fatty acids were reduced and increased, respectively, for algal cells exposed to infochemicals.

## Discussion

*Daphnia* infochemicals affect the microalga *S. subspicatus* by triggering defensive mechanisms, which include the formation of *coenobia* colonies and multi-celled aggregates (Hessen and Vandonk, 1993; Lampert et al., 1994; Lürling and vanDonk, 1996; Lürling, 1999a, b, 2003, this study). To date, the cellular processes involved in this response are not well characterized. Here, an in-depth, iTRAQ-based study was performed to identify proteins that are linked to *Daphnia*-infochemicals induced flocculation. The experimental design allowed separation of the effects of infochemicals from the ASTM effect. We further isolated the timing and mechanism of responses by examining protein expression at two stages of exposure to infochemicals, and in floc and planktonic fractions of *S. subspicatus*. Protein expression patterns induced by infochemicals at the early “alarm” (+*2 h)*, and the late “acclimation” (+*20 h)* stages of applied stress (Borowitzka, 2018) of exposure were identified, with an emphasis on the overall patterns among carbohydrates, lipids, and energy metabolism and trends in specific proteins of interest. Finally, the results were compiled to reveal any molecular mechanisms influencing flocculation.

### +*2 h* Exposure – The Alarm Phase

Following 2 h of exposure to infochemicals, protein abundance changes compared to the control flasks were observed for both the floc and the planktonic fraction of *S. subspicatus*, suggesting an increased energy requirement. For algal cells exposed to infochemicals, proteins linked to oxidative phosphorylation, providing most of the ATP needed by algae (Chen et al., 2015), were more abundant. Furthermore, there was an increase in the abundance of proteins linked to photosynthesis. In conditions where no grazer is present, algal cells would prefer to maintain their position in the upper layers of the water column, where there are more favorable conditions for growth, i.e., higher sunlight availability for photosynthesis (Lürling and van Donk, 2000). Here, the increased abundance of photosynthesis-related proteins under grazer cue conditions may, therefore, be explained by an energy-demanding diversion of algal cell metabolism to compensate for reduced access to light, due to either lower irradiance in sinking flocs or “shading” when located in inner parts of the colony.

A higher abundance of the enzyme cysteine synthase was observed for the floc fraction at +*2 h*; this is responsible for the formation of cysteine and is linked to the assimilation of sulfur (Vallon and Spalding, 2009; Shi et al., 2017). Cysteine can form disulfide bridges and therefore contributes to the structural stability of proteins. The high reactivity of the cysteine thiol group has also been linked to its role as a precursor for a variety of essential biomolecules, which have been linked to adaptive responses in changing environments. These include protection against oxidative stress, detoxification from xenobiotics and heavy metals, in addition to a defense response against herbivores and pathogens (Romero et al., 2014; Aziz et al., 2016; Shi et al., 2017). Cysteine has also been reported to stimulate bio-flocculation of bacteria by promoting the production of extracellular proteins containing more disulfide bonds (Xie et al., 2013). This study also suggested that these secreted proteins were more stable due to the presence of disulfide bonds. In our study, the higher abundance of cysteine synthase in the floc fraction may, therefore, suggest that sulfur is required for *S. subspicatus* to flocculate as a defense response to grazers’ infochemicals.

Photosynthesis is the process through which energy from light is captured to stimulate the synthesis of carbohydrates; for the floc fraction, unique DEPs linked to carbohydrates metabolism were less abundant, suggesting that algal cellular sinks might utilize the products of photosynthesis to stimulate processes other than carbohydrate accumulation. Several examples can be found in the literature for a reduction of carbohydrate metabolism in response to environmental perturbations. For example, in Shanmuganathan et al. (2004) reported that *Saccharomyces cerevisiae* cells subjected to oxidative stress showed an oxidation/inactivation of glycolytic enzymes, causing a rearrangement of glucose equivalents through the pentose phosphate pathway to provide the required reducing power, in the form of NADPH (Nicotinamide Adenine Dinucleotide Phosphate), for antioxidant defense mechanisms. Protein abundance changes related to carbohydrate metabolism for the planktonic fraction in *S. subspicatus* showed that the enzymes isocitrate dehydrogenase [NADP] and catalase were more and less abundant, respectively, compared to control conditions. Isocitrate dehydrogenases catalyze oxidative reactions which require either NAD+ or NADP+ to produce NADH and NADPH, respectively, and both act as cell protectants against oxidative damage (Kil et al., 2006). During normal cell metabolism, reactive oxygen species (ROS) are inevitably produced; these ROS increase under stress conditions and can act as signaling molecules to trigger cell responses (Michelet et al., 2013; Couto et al., 2016). The connection between ROS signaling and cellular redox have been suggested to be mediated by NADPH, among others (Mittler et al., 2011) and ROS production could be stimulated through the inhibition of the redox-sensitive enzyme catalase (Kil et al., 2006). ROS have been reported to be able to change the activity of several regulatory enzymes and in particular phosphatases like the mitogen-activated protein kinase (MAPK) phosphatases (Demidchik, 2015). In plants, ROS signaling has been linked to many other different signaling networks, including redox responses, and in some circumstances accumulation of ROS was found to either be the direct result or lead the way to signaling processes through these networks. This would be the case for the MAPK cascade (Mittler et al., 2011). We linked unique DEPs for the planktonic fraction to signal transduction, specifically to the MAPK class. Sensing of stress signals and their transduction into adaptive responses is of vital importance to adapt and survive in changing conditions. In plants, MAPK pathways are connected to the regulation of growth, development and cell division, and in response to a wide range of both abiotic and biotic stimuli, including light, temperature, salinity, ROS, or pathogen attack (i.e., damage to the cell surface) (Pitzschke et al., 2009; Livanos and Apostolakos, 2012). These results, therefore, might suggest the role of the MAPK signaling pathway in the alarm response of *S. subspicatus* to infochemicals triggering cell-division and therefore colony formation. In addition, at this stage protein abundance changes related to carbon metabolism for the floc fraction in *S. subspicatus* showed a lower abundance of RuBisCo compared to control. It has previously been reported that for some *Chlamydomonas* strains, a diminished abundance of this protein is associated with a high production of ROS and that downregulation occurs to favor energy transfer to other metabolic pathways (Johnson, 2011).

### +*20 h* Exposure – The Acclimation Phase

Protein abundance changes for the floc fraction at 20 h indicated an increased energy requirement for *S. subspicatus* in response to infochemicals; however, the concomitant decrease in the abundance of proteins involved in energy metabolisms for the planktonic fraction suggests that *S. subspicatus* cells might try to minimize energy acquisition while maintaining their colony form or alternatively divert most of their efforts to keep cells in the floc form. Furthermore, the floc fraction continued to have a higher abundance of the enzyme cysteine synthase, supporting its role in bio-flocculation. For carbohydrate metabolism, contrary to what was found at the alarm phase, the planktonic fraction showed a decreased abundance of the isocitrate dehydrogenase and hydrolases, while phosphatases were more abundant. As mentioned in the previous section, ROS are normally and inevitably produced because of cell metabolism, however, under stress conditions their production is increased, and ROS can act as signaling molecules to initiate cell responses (Michelet et al., 2013; Couto et al., 2016), modulating the activity of many regulatory enzymes including MAPK phosphatases (Demidchik, 2015). Wei et al. (2017) also reported a reduction of carbohydrate metabolism upon palmelloids formation in *Dunaliella salina* following salt stress, with proteins involved in glycolysis, the pentose phosphate pathway, starch mobilization and glucose metabolism. In that case, and in accordance to our findings, a decreased cellular carbohydrate level corresponded to an increase in extracellular carbohydrates, indicating the activation of mechanisms to sustain osmotic equilibrium between intra- and extracellular conditions.

At the acclimation phase, unique DEPs were linked to MAPK signaling cascade for the planktonic fraction again, dominated by *coenobia*, as well as for the floc fraction. In plants, MAPK pathways are involved in the regulation of cell division (Livanos and Apostolakos, 2012) and which could explain the occurrence of *coenobia* colonies. Similarly, in yeast, cell-cell adhesion can be conferred by adhesins, a special class of cell wall proteins whose synthesis is controlled by various stress-induced signaling cascades pathways, including MAPK. Relevant stress factors include limiting nutrients conditions and/or exposure to specific chemical cues such as the plant hormone indoleacetic acid (IAA) (Verstrepen and Klis, 2006). In fact, yeast cells would recognize these cues that signal the presence of the plant host and instigate the changes such as adhesion and/or filamentation (Prusty et al., 2004). It has previously been reported that the genes responsible for aggregation within yeast biofilm formation are mediated through MAPK pathways by extracellular cAMP (cyclic adenosine monophosphate) (Braun, 2008). Interestingly, the first contributor to PCA-dimension 1 at the acclimation phase is the enzyme AMPSase, involved in *de novo* AMP biosynthetic process (see Table 4). Altogether, these results suggest the role of the MAPK signaling pathway in the adaptive response of *S. subspicatus* to infochemicals, triggering and maintaining cell-division (for colony formation), and promoting flocculation (cell-cell adhesion).

Similar to the alarm phase, the acclimation stage led to protein abundance changes related to carbon metabolism for the floc fraction in *S. subspicatus*, including a lower abundance of RuBisCo compared to both the control and planktonic fraction. Only the planktonic fraction exhibited variations in protein abundance for lipid metabolism, in the form of fatty acids biosynthesis. The proteins involved, e.g., 3-oxoacyl-[acyl-carrier-protein] synthase (inferred from *Bathycoccus prasinos*) and 3-oxoacyl-[acyl-carrier-protein] reductase (inferred from *Chlamydomonas reinhardtii*) are both related to the synthesis of fatty acids (Yokoyama et al., 2001). Fatty acids are involved in multiple cell functions, including incorporation into cellular membranes (Chan and Vogel, 2010). Their characteristics affect the fluidity of the cell membrane, an essential feature for the mobility and functionality of cellular functions including the diffusion of molecules across the membrane as well as an accurate separation of membranes during cell division (Haddaji et al., 2017). Furthermore, fatty acids are involved in photosynthesis (Allakhverdiev et al., 2009) and signal transduction (Graber et al., 1994). FAMEs quantification and characterization data in this study showed that *S. subspicatus* cells responded to infochemicals with an increase in the overall amount of intracellular fatty acids produced and with a redistribution of their composition, with varying lengths of acyl chains and different degrees of saturation (Figures 8A,B). The composition of fatty acids is reported to change with changing environmental conditions to allow algal cells to cope with varying circumstances or triggering defense responses (Wacker et al., 2016; Darki et al., 2017), with their function being determined by length, position, and saturation level of their acyl chain(s) (Walley et al., 2013). Although small, the presence of fatty acids in the sEPS matrix of *S. subspicatus* cells exposed to *Daphnia* infochemicals was a meaningful indicator of an investment of the algal cells into the production and release of defense compounds. In fact, some PUFAs act as a defense mechanism in diatoms against grazers and bacteria, deterring feeding or impairing their growth or reproduction (Ianora et al., 2004; Amin et al., 2012).

### The Effect of ASTM

The addition of ASTM water alone induced protein abundance variations in *S. subspicatus* cells, a response which was largely unexpected. Among the unique DEPs, we noted the presence of heat shock proteins which are linked to salt stress response for algae and plants. Pandhal et al. (2009a, b) investigated the molecular adaptation mechanism against salinity stress of the cyanobacteria *Synechocystis* sp. PCC 6803 and *Euhalothece* sp. BAA001 respectively, to report an increase in the abundance of heat shock proteins 70s (HSP70). These are molecular chaperons which have an essential role in the protection of algal/plant cells through correct protein folding. Our results would suggest even low salt concentrations (MgSO_4_⋅7H_2_O: 0.25 g/L; NaHCO_3_: 0.19 g/L; KCl: 0.004 g/L and CaSO_4_⋅2H_2_O: 0.12 g/L), elicit metabolic responses in *S. subspicatus* cells, which are different from the protein abundance variations occurring in the presence of infochemicals. The presence of infochemicals might alleviate the effects of salinity on *S. subspicatus*, similar to what was reported for the jasmonates signaling compounds, which mediate defense mechanisms in plants against herbivores and attenuate salinity stress (Dar et al., 2015). We suggest that future research should be directed toward the evaluation of the interference of salts in the infochemicals induced response in *S. subspicatus.*

### Mechanisms of Infochemicals-Induced Flocculation

Our proteomics data indicates that when colony formation and aggregation of *S. subspicatus* occurs in response to *Daphnia* infochemicals at the +*2 h* alarm phase, increased energy resources are required, while not affecting algal growth. A key role is envisaged for the synthesis of cysteine, a primary amino acid, a precursor of defense biomolecules and a promoter of bio-flocculation through the production of extracellular proteins with disulfide bonds. Higher abundance of proteins related to photosynthesis, coupled with decreased protein abundance for carbohydrate metabolism, suggested bio-flocculation is also boosted by the export of carbohydrates in the sEPS matrix. Moreover, an investment by microalgal cells into the production and release of defense compounds in the forms of fatty acids could constitute a large part of the ‘glue’ responsible for holding algal cells together. The data also suggested infochemicals induced flocculation may be sustained through MAPK signaling cascades. It remained important to distinguish between aggregation and colony formation and the proteomic experimental results, contrasting floc and planktonic cell responses, support this idea that there are two separate processes. In fact, in contrast to aggregation, colony formation required higher energy demands at the alarm phase which later decreased at the acclimation stage, therefore suggesting a trade-off between colony formation and supporting the floc formation. Results suggested a role of fatty acid metabolism in the process of colony formation, as they contribute to a variety of cellular functions, including the accurate separation of membranes during cell division.

## Conclusion

This work represents the first study unraveling the molecular processes behind the response of *S. subspicatus* to produce colonies and flocculate as an adaptive response to *Daphnia* infochemicals. These were linked to photosynthesis, carbohydrate, and lipid metabolism in addition to acting as signal transduction pathways. This is particularly valuable within the fields of ecology and evolution. From a biotechnology perspective, infochemicals could be potentially used to promote flocculation in large scale cultivation systems (e.g., open raceway ponds), inducing defensive morphological changes in microalgae. Although the direct addition of purified biological infochemicals or preparation of extracts could provide a more sustainable option than using metal salts, it would be necessary to account for their additional production costs, as well as the potential to reuse water. Similar to auto-flocculation, however, the biomass would not be contaminated with info-chemical induced harvesting. Future research should be directed toward matching the existing mass spectra to an up-to-date, annotated proteome database for this specific microalgal species to improve the number of proteins quantified. Together with analysis of the membrane proteome, this would provide a more global view of the induced cellular responses. Finally, targeting quantitation of metabolites within some of the highlighted pathways could provide further mechanistic insight.

## Data Availability Statement

The datasets generated for this study can be found in the ProteomeXchange Consortium via the PRIDE partner repository (Dataset identifier: PXD014153).

## Author Contributions

SR, AB, and JP conceived the project. SR, AB, JP, NC, and EK designed the experimental plan. SR, NC, and EK carried out the iTRAQ experiments and the sEPS extraction. SR, EK, NC, RK, JM, and TB carried out the proteins, carbohydrates, and fatty acids analyses. SR and EH carried out the flow cytometry analysis. SR carried out the data analysis and interpretation with inputs from AB, NC, EK, and JP. SR, AB, and JP wrote the manuscript. All authors have read and approved the final manuscript.

## Conflict of Interest

The authors declare that the research was conducted in the absence of any commercial or financial relationships that could be construed as a potential conflict of interest.
